# Red Wine Polyphenol Compounds Favor Neovascularisation through Estrogen Receptor α-Independent Mechanism in Mice

**DOI:** 10.1371/journal.pone.0110080

**Published:** 2014-10-09

**Authors:** Matthieu Chalopin, Raffaella Soleti, Tarek Benameur, Angela Tesse, Sébastien Faure, Maria Carmen Martínez, Ramaroson Andriantsitohaina

**Affiliations:** 1 INSERM U1063, Stress Oxydant et Pathologies Métaboliques, Angers, France; 2 Centre Hospitalier Universitaire d’Angers, Angers, France; Inserm, France

## Abstract

Red wine polyphenol compounds (RWPC) exert paradoxical effects depending on the dose on post-ischemic neovascularisation. Low dose RWPC (0.2 mg/kg/day) is pro-angiogenic, whereas high dose (20 mg/kg/day) is anti-angiogenic. We recently reported that the endothelial effect of RWPC is mediated through the activation of a redox-sensitive pathway, mitochondrial biogenesis and the activation of α isoform of the estrogen receptor (ERα). Here, we investigated the implication of ERα on angiogenic properties of RWPC. Using ovariectomized mice lacking ERα treated with high dose of RWPC after hindlimb ischemia, we examined blood flow reperfusion, vascular density, nitric oxide (NO) production, expression and activation of proteins involved in angiogenic process and muscle energy sensing network. As expected, high dose of RWPC treatment reduced both blood flow and vascular density in muscles of mice expressing ERα. These effects were associated with reduced NO production resulting from diminished activity of eNOS. In the absence of RWPC, ERα deficient mice showed a reduced neo-vascularisation associated with a decreased NO production. Surprisingly in mice lacking ERα, high dose of RWPC increased blood flow and capillary density in conjunction with increased NO pathway and production as well as VEGF expression. Of particular interest is the activation of Sirt-1, AMPKα and PGC-1α/β axis in ischemic hindlimb from both strains. Altogether, the results highlight a pro-angiogenic property of RWPC via an ERα-independent mechanism that is associated with an up-regulation of energy sensing network. This study brings a corner stone of a novel pathway for RWPC to correct cardiovascular diseases associated with failed neovascularisation.

## Introduction

Angiogenesis, the sprouting of new blood vessel, is crucial for all tissue growth, expansion and repair [1]. Angiogenesis is a multi-step process, starting with endothelial cell activation, vasodilatation and increased vascular permeability, followed by endothelial cell migration and proliferation into the perivascular space. Finally, endothelial cells assemble to form a new vessel [2]. This process occurs in physiological states as embryogenesis or after injury as well in pathological issues, such as cancer growth [3], rheumatoid arthritis [4] or diabetic retinopathy [5].

Numerous epidemiological studies have consolidated the idea that a moderate consumption of red wine is associated with a reduced cardiovascular risk [6]. The beneficial effects of red wine are reported due to its polyphenol components (RWPC). We have recently reported that the alpha isoform of the estrogen receptor (ERα) is one of the targets involved in vascular effects of RWPC [7]. Both RWPC, and delphinidin, an anthocyanin that possesses the same pharmacological profile than the total extract, interact directly with activator site of ERα inducing the activation of ERK/Src/eNOS pathway leading to endothelial NO release and then, vasodilation [7].

RWPC are reported to possess anti-angiogenic properties. In a swine study of chronic ischemia, high dose of resveratrol (100 mg/kg/day) inhibited new vessel formation, probably through an increased expression of angiostatin and thrombospondin leading to decreased expression of VE-cadherin and other pro-angiogenic factors [8]. In a rat model of peripheral ischemia, we have found that low doses (0.06 mg/kg/day) of delphinidin have no effect on blood flow recovery, however higher doses (0.6 mg/kg/day) have anti-angiogenic effects as evidenced by impaired blood flow reperfusion and decreased vascular density in the ischemic leg [9]. In an *in vivo* model of avascular rabbit cornea, quercetin (100 µM) inhibited VEGF-induced endothelial cell functions and angiogenesis and prevented VEGF-induced ERK1/2 phosphorylation [10]. On the other hand, RWPC protect against deleterious effects of cardiac and cerebral ischemia [11], [12], the correction of which requires pro-angiogenic properties to produce new blood vessels in order to rescue the infarcted area. These dual effects of RWPC remain unexplained. We have highlighted a dose-dependent effect of polyphenols in an *in vivo* model of angiogenesis triggered by peripheral ischemia to explain this paradoxical effect of RWPC [9]. A low dose of RWPC promoted angiogenesis by activation of NO, Akt/PI3K and p38 MAPK pathways, and increasing VEGF expression without altering either MMP activity or NF-κB expression. In contrast, a high dose of RWPC reduced angiogenesis *via* an inhibition of both NO/VEGF and Akt/PI3K pathways, whereas MMP activity was reduced in association with phospho-p38 and NF-κB expression [9].

Beside, RWPC activate Sirtuin 1 (Sirt-1), 5′ adenosine monophosphate-activated protein kinase (AMPKα) and peroxisome proliferator-activated receptor gamma coactivator-1 (PGC-1α) which are implicated in energy expenses and angiogenesis [13]–[17]. The mechanisms involved in angiogenic effects of RWPC have already been described in a number of studies, but the molecular target is still not fully understood. This study was designed to assess the role played by ERα in paradoxical angiogenic effects of RWPC. Using ovariectomized genetically modified mice lacking ERα fed chronically with high dose of RWPC, we first studied blood flow recovery after surgical hindlimb ischemia. Then, we evaluated underlying angiogenic mechanisms in hindlimb muscles, through analysis of vascular density, NO production, as well as expression and activation of proteins involved in angiogenic process and muscle energy sensing network.

## Methods and Materials

### Products

RWPC was obtained from Société Française des Distilleries (Vallon Pont d’Arc, France). The composition is, in mg/g of dry powder: 480 proanthocyanidins, 61 total anthocyanins, 19 free anthocyanins, 38 catechin, 18 hydroxycinnamic acids, 14 flavonols and 370 polymeric tannins.

For animal feeding, RWPC was dissolved in water and given by daily gavage.

### Ethics Statement

The procedures followed in the care and euthanasia of the study animals was in accordance with the European Community standards on the care and use of laboratory animal and was approved by the Ethical Committee for Animal Research of Angers University. The animals were housed in a regulated environment with a constant ambient temperature of 24°C. They had free access to standard laboratory food and water.

### Animals

Twelve-week-old female ERα Wild Type (WT) or Knock Out (KO) mice were ovariectomized. After 7 days, mice were treated for 28 days with RWPC (20 mg/kg/day) or water (n = 5 animals for each group). One day after beginning of treatment, mice were anesthetised with isoflurane and underwent surgery to induce unilateral hindlimb ischemia. The ligature was performed on the left femoral artery proximal to the bifurcation to the saphenous and popliteal arteries, as previously described [9], [18]. After 7, 14, 21 and 28 days of ligature, blood flow was measured as described below. At 28 day, mice were euthanized and tissues were sampled for biochemical and histological analysis.

### Quantification of perfusion: Laser-Doppler Blood Flow (LDBF) analysis

In order to provide a functional evidence of ischemia, laser doppler perfusion imaging was performed in anesthetized mice, as previously described [18]. Animals were settled on a heating plate to maintain a stable cutaneous temperature in order to minimize temperature variation throughout the experiments. Leg perfusion was then measured using a Laser Doppler flow probe (PF 408, Perimed, Stockholm, Sweden). Blood flow was recorded during ∼3 min. At least 2 flow measurements were performed per leg. Blood flow perfusion was expressed as a ratio of left (ischemic) to right (non-ischemic) leg, as described by Limbourg et al. [19].

### Vascular density

Vascular density, as an index of neovascularisation, was examined by counting the number of vessels taken from the ischemic and non-ischemic limbs. Ischemic and non-ischemic gastrocnemius muscles were dissected and embedded in Tissue-Tek O.C.T (Sakura Finetek, Zoeterwoude, The Netherlands). Cryosections (7 µm) were fixed (5 min at −20°C) in 100% methanol, and saturated (1 h at room temperature) in blocking buffer (5% non fat dry milk in PBS and 0.05% Tween 20). Fixed and blocked tissue sections were incubated overnight at 4°C with rat anti-mouse CD31 antibody (1:100, BD Biosciences, San Jose, CA). After three washes, tissue sections were incubated (1 h at room temperature) with goat anti-rat IgG fluorescein-conjugated (1:100, Southern Biotech, Birmingham, AL) to identify vessels as described by Limbourg et al. [19]. After final washes, sections were mounted on glass slides. MRC-1024ES confocal equipment mounted on a Nikon Eclipse TE 300 inverted microscope was used for the optical sectioning of the tissue. Digital image recording was performed using the Laser Sharp Software. Vessels were quantified using ImageJ software and counted in at least four randomly selected fields for each muscle section, and the mean value for each section was calculated (magnification x40).

### NO spin trapping and electron paramagnetic resonance (EPR) studies

Detection of NO production was performed using the technique with Fe^2+^ diethyldithiocarbamate (DETC, Sigma-Aldrich) as spin trap. Briefly, after treatment, aortas, ischemic or non-ischemic gastrocnemius and soleus muscles were dissected for NO production measurement by their incubation for 30 min in Krebs-Hepes buffer containing: BSA (20.5 g/L), CaCl_2_ (3 mM) and L-Arginine (0.8 mM). NaDETC (3.6 mg) and FeSO_4_-7H_2_O (2.25 mg, Sigma-Aldrich) were separately dissolved under nitrogen gas bubbling in 10 ml volumes of ice-cold Krebs-Hepes buffer. The solutions were rapidly mixed to obtain a pale yellow-brown opalescent colloid Fe(DETC)_2_ solution (0.4 mM), which was used immediately to incubate organs for 45 min at 37°C. After incubation spin trap was removed, organs were immersed in physiological salt solution and frozen in liquid nitrogen.

NO measurement was performed on a table-top x-band spectrometer Miniscope (Magnettech, MS200, Berlin, Germany). Recordings were made at −196°C, using a Dewar flask. Instrument settings were 10 mW of microwave power, 1 mT of amplitude modulation, 100 kHz of modulation frequency, 150 s of sweep time and 5 scans. Signals were quantified by measuring the total amplitude, after correction of baseline as done previously [20].

### Western Blotting

After 28 days of treatment, mice were sacrificed and gastrocnemius and soleus muscles from ischemic and non ischemic hindlimb were cut out and were homogenized and lysed. Proteins (80 µg) were separated on 10% SDS-PAGE electrophoresis gel. Blots were probed with eNOS, phospho-eNOS Ser 1177, phospho-eNOS Thr 495, cav-1, phospho-cav-1, NF-κB, VEGF, Sirt-1, PGC-1α/β and AMPKα antibodies (1:500, Cell Signalling, Danvers, MA). A polyclonal anti-mouse ß-actin antibody (1:2000, Sigma-Aldrich) was used to visualize protein gel loading. Bound antibodies were detected with a secondary peroxidase-conjugated anti-rabbit or anti-mouse IgG (Promega, Charbonnieres, France). The blots were visualized using the enhanced chemiluminescence system (ECL Plus, Amersham Biosciences, Piscatawat, NJ) or for phosphorylated proteins using the Super Signal West Femto (Thermo Scientific Pierce, Brebières, France) and quantified by densitometry and normalized to β-actin expression. Results are expressed with ischemic/non ischemic ratio for each protein.

### Data analysis

Data are represented as mean ± SEM, n represents the number of experiments. Statistical analyses were performed by a one way analysis of variance (ANOVA), and Mann-Whitney U tests or ANOVA for repeated measures and subsequent Bonferroni post hoc test. P<0.05 was considered to be statistically significant.

## Results

### Laser doppler quantification of perfusion in the hindlimb

**Figure 1:**
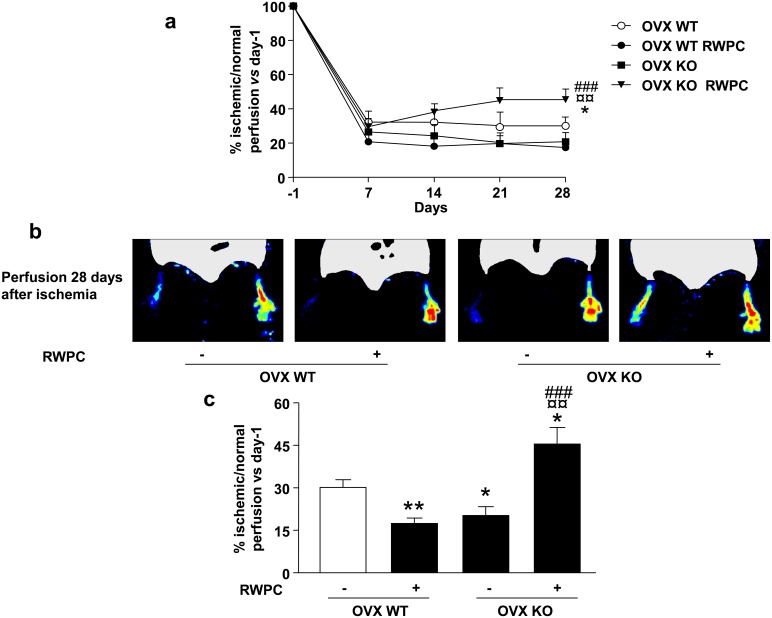
Evaluation of leg neovascularisation after femoral artery ligature in ovariectomized (OVX) ERα WT and KO mice treated or not with 20 mg/kg/day of red wine polyphenol compounds (RWPC). (a) Quantification of blood perfusion at different times (one day before ligature (day-1), and days 7, 14, 21 and 28 after ligature) in four groups of mice (n = 5/group). Values are expressed in mean ± SEM as the ischemic/non ischemic leg ratio *vs* day-1. (b) Blood flow perfusion (n = 5/group) with typical images. (c) Quantification of perfusion. Values are expressed in mean ± SEM as the ischemic/non ischemic leg ratio *vs* day-1. *P<0.05, **P<0.01 vs control OVX WT; ¤¤P<0.01 vs OVX WT treated; ###P<0.001 vs control OVX KO.

Evaluation of hindlimb blood flow perfusion was performed weekly in both ischemic and non-ischemic leg, by laser doppler blood flow analysis. Ischemia was induced by ligature and excision of the left femoral artery, as previously described [18]. No difference could be observed in the degree of post-operative ischemia between the four groups of mice. Seven days after ligature, foot blood flow was significantly lower in the ischemic than in the non-ischemic leg, but no differences were found in blood flow recovery between the four groups (Fig. 1a). Fourteen and 21 days after ligature, only ERα KO mice treated with RWPC displayed higher blood flow recovery in comparison with other groups (Fig. 1a). At day 28 after ligature in WT mice, RWPC decreased by 0.57-fold the ischemic/non-ischemic blood flow ratio (P<0.01) compared with control. Deletion of ERα leaded to a reduced perfusion by 0.7-fold the ischemic/non-ischemic blood flow ratio *vs* control (P<0.05). Surprisingly in KO mice, RWPC induced a greater blood flow perfusion compared to untreated KO mice (Fig. 1a–c). This increase in blood flow recovery was also greater than that obtained in WT mice (P<0.01) with or without RWPC treatment (P<0.05).

### Vascular density

**Figure 2:**
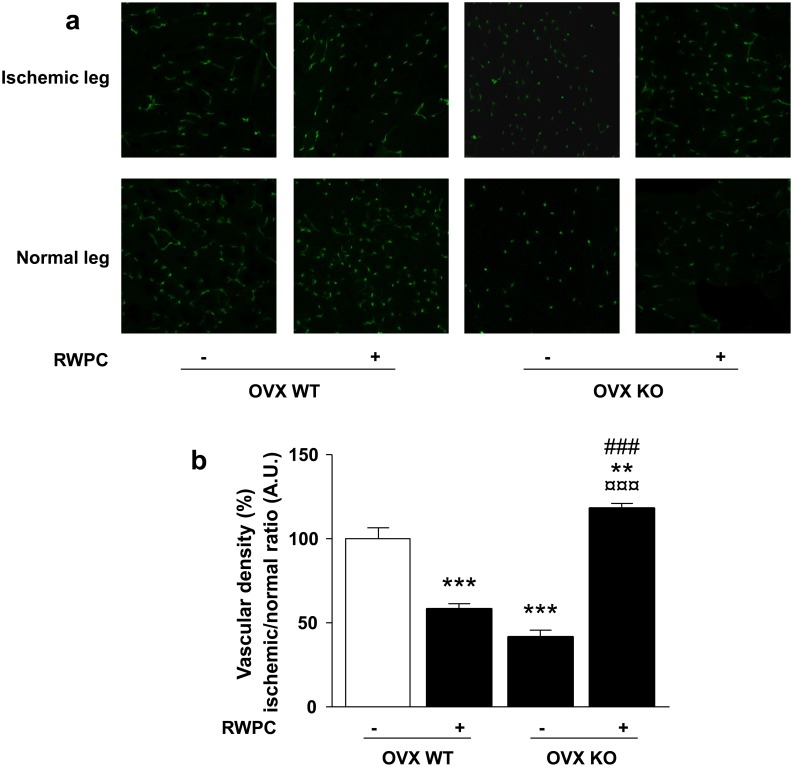
Evaluation of capillary density in ovariectomized (OVX) ERα WT and KO mice treated or not with 20 mg/kg/day of RWPC for 28 days. (a) Typical images of capillary density by CD31 staining in ischemic and normal legs. (b) Quantification of capillary density. Values are expressed in mean ± SEM as the ischemic/non ischemic leg ratio (n = 5). **P<0.01, ***P<0.001 vs. control OVX WT; ¤¤¤P<0.001 vs OVX WT treated; ###P<0.001 vs control OVX KO.

Data from the laser doppler analysis were confirmed by vascular density measurement using a fluorescent CD31 staining visualized by confocal microscopy. Twenty eight days after ligature, RWPC decreased the vascular density expressed as ischemic/normal ratio by 0.58-fold (P<0.001) compared with the control WT mice. In mice lacking ERα, vascular density was reduced by 0.42-fold (P<0.001) in comparison with WT mice. Interestingly, RWPC treatment in KO mice induced an increase in vascular density compared to WT mice (1.18-fold; P<0.01) as well as WT treated mice (2.02-fold; P<0.001) and control KO mice (2.82-fold; P<0.001) (Fig. 2).

Altogether, we report that the anti-angiogenic ability of RWPC requires the presence of ERα. It is also found a pro-angiogenic activity of RWPC via a mechanism independent of the presence of ERα.

### NO production in aorta and hindlimb muscle

**Figure 3:**
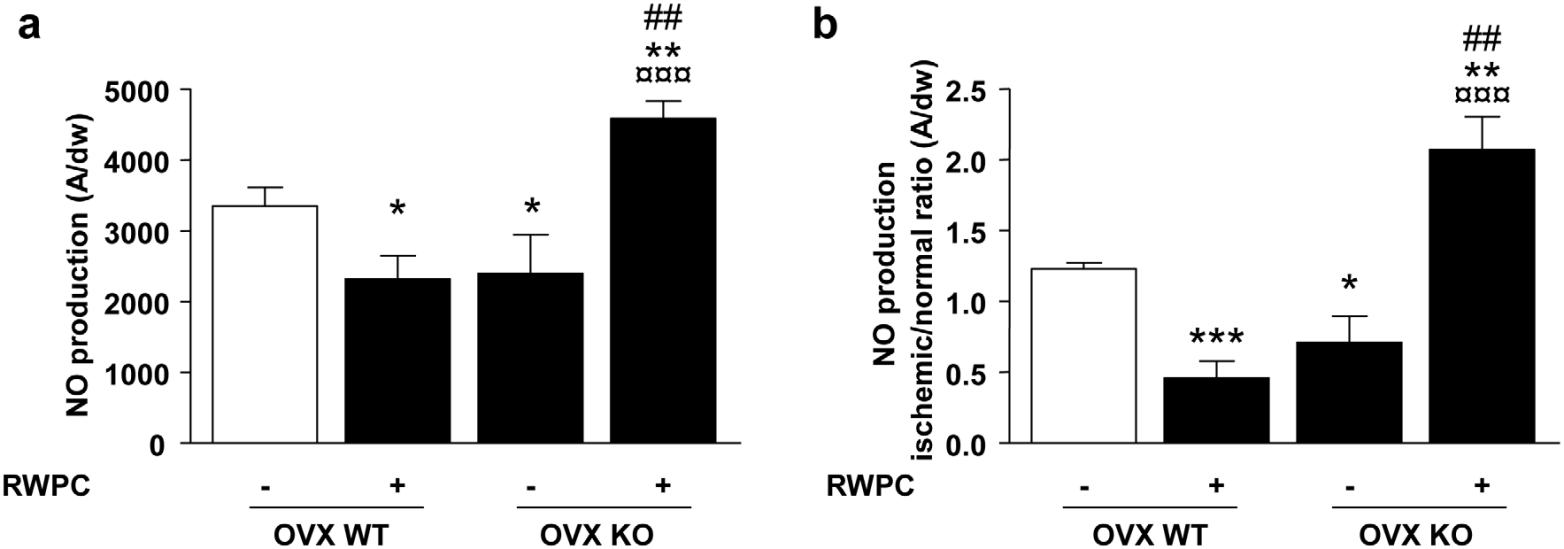
Quantification of the amplitude of NO signals in aorta (a) and in hindlimb muscles (b) from ovariectomized (OVX) ERα WT and KO mice treated or not with 20 mg/kg/day of RWPC for 28 days. Values are expressed as amplitude/mg of dried weight (dw) of aorta or as ratio of amplitude/mg of dw of muscle (mean ± SEM) (n = 5). *P<0.05, **P<0.01, ***P<0.001 vs. control group; ¤¤¤P<0.001 vs OVX WT treated; ###P<0.001 vs control OVX KO.

NO production was assessed both in the aorta as a control regarding a non-ischemic tissue (Fig. 3a) and in the ischemic skeletal muscle (Fig. 3b). As expected, RWPC significantly reduced NO production in the aorta from WT mice (0.7-fold; P<0.05). Also, deletion of ERα decreased the NO production in aorta (0.7-fold; P<0.05). Interestingly, RWPC significantly enhanced NO production in aorta from ERα deficient mice compared either from control WT (0.7-fold; P<0.01), RWPC-treated WT mice (1.9-fold; P<0.001) or control KO (1.9-fold: P<0.001).

In skeletal muscle from WT mice, RWPC decreased significantly NO production compared to control (2.6-fold; P<0.001). Mice lacking of ERα displayed a NO level significant lower than WT (1.7-fold; P<0.05). Surprisingly, RWPC significantly increased NO production in ERα deficient mice compared either to control WT (1.7-fold; P<0.01), RWPC-treated WT mice (4.5-fold; P<0.001) or control KO (2.9-fold; P<0.01). These data suggest that NO level of hindlimb ischemia was correlated to the changes in blood flow in all groups of mice studied.

### Effects of ERα deletion and RWPC in non ischemic muscles

**Figure 4:**
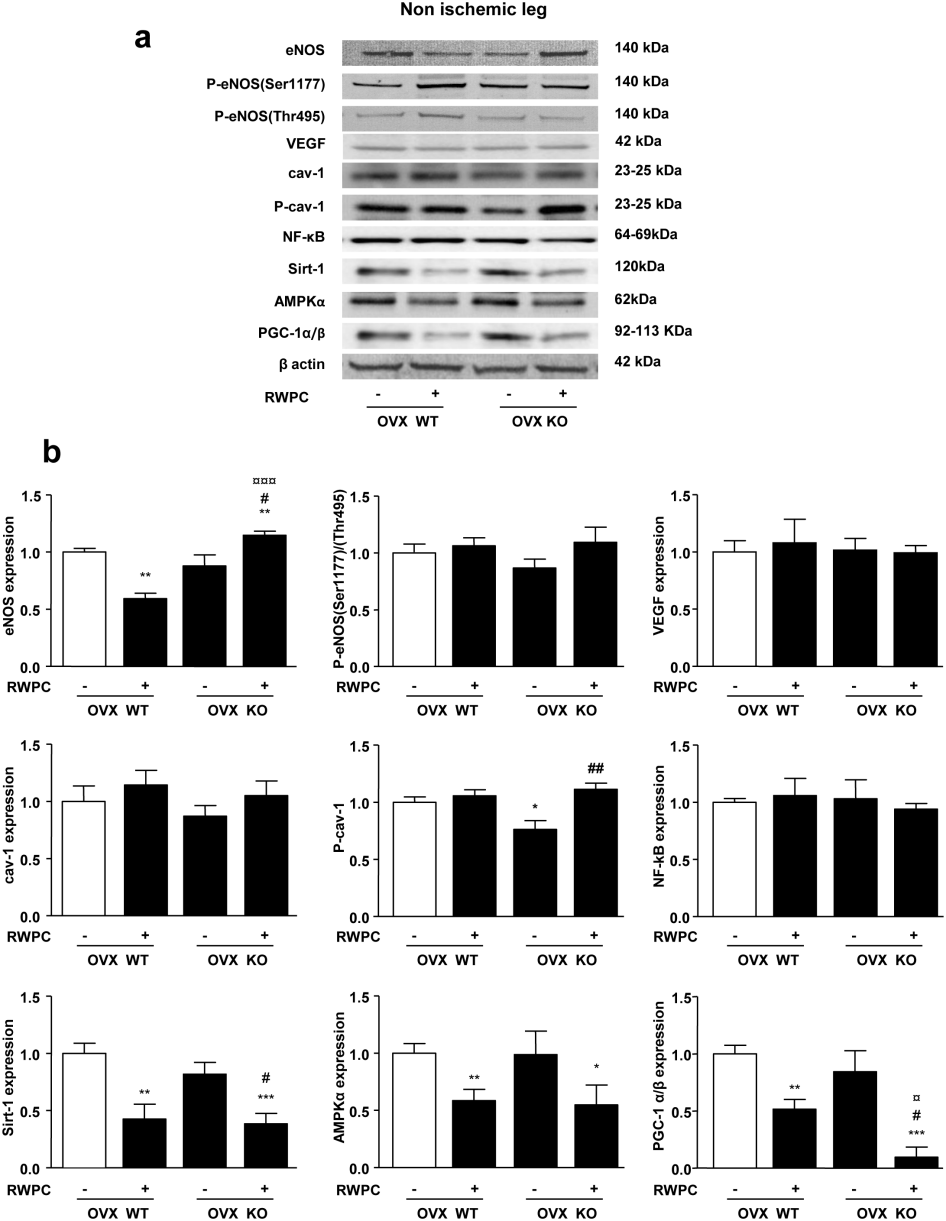
Evaluation of the protein expression levels extracted from the right (non ischemic) gastrocnemius and soleus muscles in ovarectomised (OVX) ERα WT and KO mice treated or not with 20 mg/kg of RWPC for 28 days. Representative images of blots showing protein expression levels in non ischemic leg muscle (a) Quantification of protein expressions expressed normalized to β-actin expression, (b) (mean ± SEM) (n = 5). *P<0.05, **P<0.01, ***P<0.001 vs. control group; ##P<0.01; ###P<0.001 vs control OVX KO; ¤P<0.05, ¤¤P<0.01, ¤¤¤P<0.001 vs OVX WT treated.

The impact of ERα deficiency and RWPC on signalling pathways in non-ischemic hindlimb muscles has been evaluated. As shown in Figure 4, among the proteins evaluated, deletion of ERα decreased only phosphorylation of cav-1 in comparison with WT mice normalized to β-actin expression. In the present study, we also found that RWPC decreased Sirt-1/AMPKα/PGC-1α/β expressions in the non ischemic area in both WT and KO mice. It might be possible that *in vivo* ischemic conditions are need for RWPC to activate these pathways, and such conditions are not fulfilled in the non ischemic area.

### Effect of RWPC on proteins in ischemic/non-ischemic skeletal muscle ratio: role of ERα

**Figure 5:**
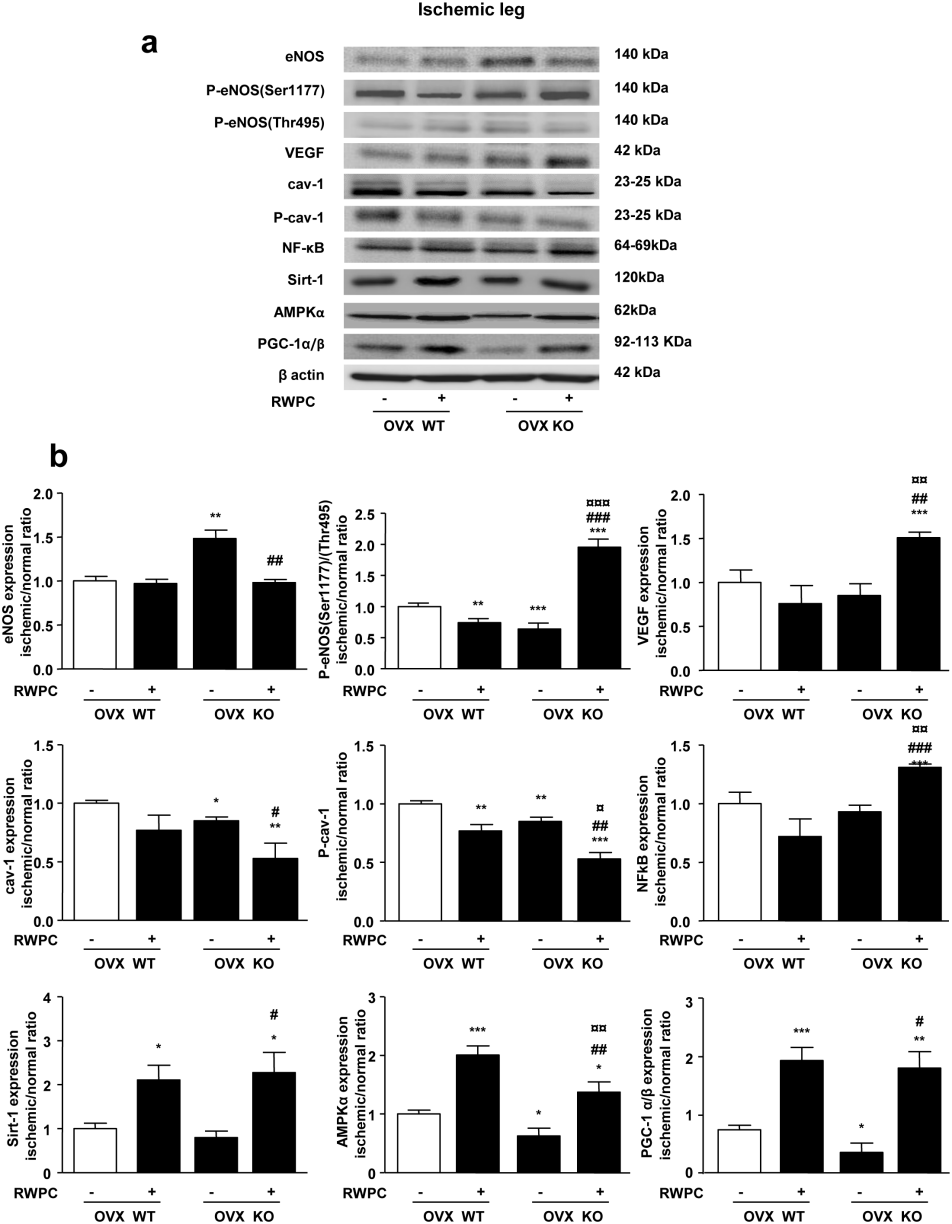
Evaluation of the protein expression levels extracted from the right (normal) and left (ischemic) gastrocnemius and soleus muscle in ovarectomised (OVX) ERα WT and KO mice treated or not with 20 mg/kg of RWPC for 28 days. Representative images of blots showing protein expression levels in ischemic leg muscle (a) Quantification of protein expressions expressed as a ratio of normal/ischemic protein expression in comparison with WT control group normalized to β-actin expression, (b) (mean ± SEM) (n = 5). *P<0.05, **P<0.01, ***P<0.001 vs. control group; ##P<0.01; ###P<0.001 vs control OVX KO; ¤P<0.05, ¤¤P<0.01, ¤¤¤P<0.001 vs OVX WT treated.

As shown in Figure 5 (a, b), in skeletal muscles from WT mice, although RWPC did not modify eNOS expression, they decreased eNOS activity as revealed by a reduced phosphorylation on its activator site (Ser1177) without changes in phosphorylation on its inhibitor site (Thr495). Also, RWPC decreased cav-1 phosphorylation without affecting its expression. All these modifications are consistent with observed decreased NO production. In muscles taken from ERα deficient mice, increased eNOS expression was associated with enhanced phosphorylation on Thr495 and decreased cav-1 expression and phosphorylation, leading to decreased activity and reduced NO production. In mice lacking ERα, treatment with RWPC induced an increased activity of eNOS due to its phosphorylation on Ser1177 associated with decreased cav-1 expression and activation, which is in agreement with the detected NO level in this tissue. Whereas RWPC were not able to significantly modify expression of VEGF and NF-κB in WT mice, RWPC increased expression of both proteins in KO mice. RWPC treatment enhanced expression of AMPKα and PGC-1α/β in tissues taken from WT mice. Deletion of ERα was associated with a decreased expression of, AMPKα and PGC-1α/β, in hindlimb muscles when compared to WT mice. However, RWPC treatment enhanced expression of Sirt-1, AMPKα and PGC-1α/β in tissues taken from ERα deficient mice in a similar extent than WT mice.

These data suggest that RWPC exerted their anti-angiogenic property in presence of ERα by decreasing NO production. More interesting, we describe for the first time that the pro-angiogenic effect of RWPC resulting from activation of eNOS is associated with increased Sirt-1, AMPKα and PGC-1α/β pathways independently from ERα.

## Discussion

The present study shows that, after peripheral ischemia, deletion of ERα leads to reduced neovascularisation associated with a decreased of NO production, diminution of eNOS activity, and AMPKα and PGC-1α/β expressions. In addition, the described results confirm that high dose of RWPC reduces neovascularisation in mice subjected to hindlimb ischemia via inhibition of NO pathway despite their ability to increase Sirt-1, AMPKα and PGC-1α/β expressions. Interestingly, they also demonstrate that deletion of ERα unmasks pro-angiogenic activities of RWPC in association with increased NO/VEGF pathways and NF-κB without affecting their ability to activate Sirt-1/AMPKα/PGC-1α/β axis. Thus, NO/VEGF, NF-κB and Sirt-1/AMPKα/PGC-1α/β axis plays a role in the pro-angiogenic effect of RWPC via an ERα-independent mechanism.

We have previously shown that in the rat model of induced peripheral ischemia, the same dose of RWPC used in the present study, had anti-angiogenic properties via inhibition of NO pathway and NF-κB expression [9]. Similar effects are observed in the present study in WT mice, confirming that reduced angiogenesis induced by high doses of RWPC involves of NO/VEGF and Akt/PI3K pathways and reduction of MMP activity, phosphorylation of p38 and NF-κB expression. In agreement with these results other authors have shown that a higher dose of RWPC (100 mg/kg/day) prevents the expression of pro-angiogenic factors [21], including VEGF and eNOS most likely by inhibiting oxidative stress. As previously reported, the molecular identity of the compounds responsible for the anti-angiogenic properties of high dose of RWPC is not fully known. However, delphinidin one the major compounds found in RWPC, inhibited angiogenesis in a rat model of hindlimb ischemia [9]. Thus, this compound displays the same pharmacological profile than the total extract. Indeed, our group has shown that delphinidin inhibits *in vitro* and *in vivo* angiogenesis. In human umbilical vein endothelial cells the anti-proliferative properties of delphinidin involves cyclin D1- and A-dependent pathway [22]. Furthermore, in chicken embryo chorioallantoic membrane assay, delphinidin is able to decrease capillary development through an ERK1/2 phosphorylation, independent of NO pathway and correlated with suppression of cell progression by blocking the cell cycle in G0/G1 phase in bovine aortic endothelial cells [23]. Altogether, further studies are needed to sort out the molecular identities of RWPC for its anti-angiogenic effect that might explain some of the differential mechanisms found compared to delphinidin.

It has been shown that high (20 mg/kg *in vivo*, 10^−2 ^g/l *in vitro*) or low (0.2 mg/kg *in vivo*, 10^−4 ^g/l *in vitro*) RWPC increase endothelial NO production [7], [20], [24]–[27] via a NADPH oxidase sensitive pathway and the activation of the ERα. Very recently, we found that low concentration of RWPC induces *in vitro* angiogenesis by a mechanism sensitive to ER inhibitor involving both NADH oxydase and NO pathways associated with increased mitochondrial capacity [27]. Also, ERα agonists promote angiogenesis in human myometrial microvascular endothelial cells through the activation of NO and VEGF pathways [28]. Estrogens also affect positively angiogenesis by inducing mobilization and recruitment of endothelial progenitor cells [28]. In the present study, deletion of ERα decreased NO production via reduced eNOS activity and reduced AMPKα and PGC-1α/β expressions. In conjunction with this study, Jesmin et al. [29] have previously reported disrupted levels of VEGF associated with decreased capillary density in ERα KO mice. Altogether we reinforce the notion that ERα plays a role in neovascularisation after peripheral ischemia. As previously indicated, the identification of molecular compounds which elicit the effects reported in the present study has not been carried out. Nevertheless, it was found that low and high doses of RWPC are adequate to produce a sufficient circulating concentration of compounds able to modulate angiogenesis [9]. The latter study support the hypothesis of different doses of RWPC modulate the balance between circulating pro- and anti-angiogenic compounds that might determine the final effect of RWPC *in vivo*.

In addition to the effects of RWPC mentioned above in WT mice, we highlight a pro-angiogenic activity of high dose of these compounds in mice lacking ERα. These properties were associated with increased NO production through the induction of NO and VEGF pathways. Interestingly, high dose of RWPC enhanced Sirt-1, AMPKα and PGC-1α/β expressions in hindlimb ischemia of both wild type and ERα-deficient mice but paradoxically reduced and favoured neovascularisation in WT and KO mice, respectively. Sirt-1, AMPKα and PGC-1α/β have been reported to play a role in angiogenesis. Sirt-1, a class III histone deacetylase, is involved in multiple physiological processes [30] and is activated under stress condition to regulate cell cycles [31]. Interestingly, Sirt-1 is able to upregulate eNOS, reduces smooth muscle cells senescence and suppresses ROS and inflammation in arteries [32]. Sirt-1 activation by laminar flow and statin treatment also increases eNOS activity and NO production [33], [34]. Thus, the interaction between Sirt-1 and eNOS might contribute to the pro-angiogenic properties of RWPC. Indeed, Sirt-1 is involved in angiogenic process, as mice displaying endothelial cell-specific deletion of *SIRT1* present an impaired angiogenesis in response to ischemia due to its ability to deacetylate forkhead transcription factor Foxo1, an essential negative regulator of blood vessel development, to restrain its anti-angiogenic activity [35]. Sirt-1 may inhibit angiogenesis by suppressing hypoxia inducible factor 1 alpha activity [36]. AMPKα is implicated for angiogenesis process in endothelial cells from human umbilical vein [37]. AMPKα can also mediate VEGF-induced NO production in human aortic endothelial cells [38]. Finally, mice lacking PGC-1α fails to recover blood flow after ischemia [39] and are associated with a decreased capillary density and VEGF expression [40]. Interaction between these three molecular targets of RWPC has been reported in the literature [41]. Altogether, one can advance the hypothesis, that the ERα-independent pathway activated by RWPC is associated to the activation of Sirt-1/AMPKα/PGC-1α/β axis leading to an increased NO production and therefore participate to enhance neovascularisation under the experimental condition used. Therefore, some of RWPC are pro-angiogenic being able to activate Sirt-1/AMPKα/PGC-1α/β cascade. However, the presence of ERα hinders this effect by acting on VEGF/NO axis as well as NF-κB pathway. Thus, repression of these pro-angiogenic pathways became predominant and conduced led to failed angiogenesis at high dose of RWPC.

**Figure 6:**
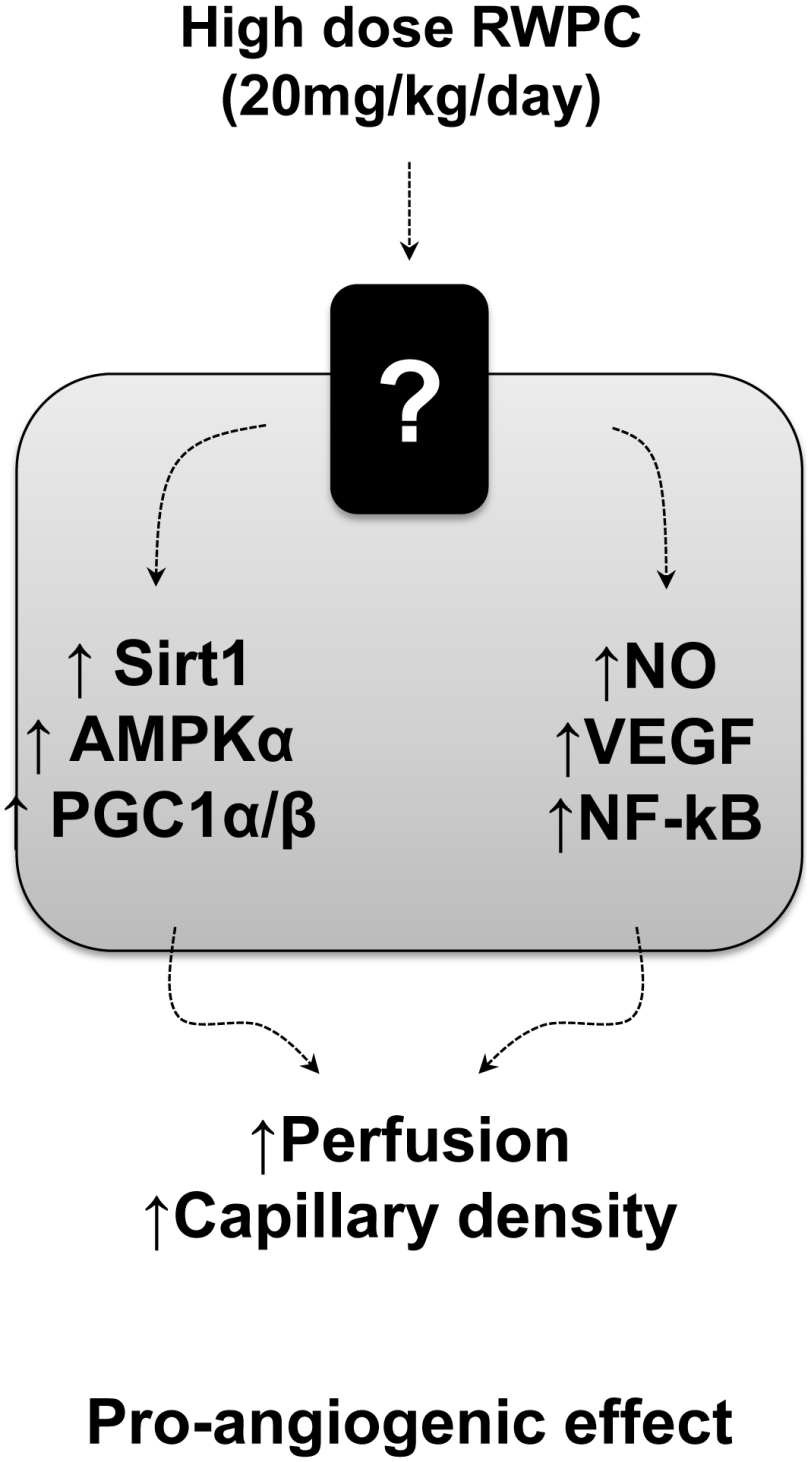
Scheme of pro angiogenic effect of RWPC. Deletion of ERα unmask the ability of RWPC to enhance neovascularisation VEGF/NO/NF-kB and Sirt-1/AMPKα/PGC-1α/β pathways. These effects concur to increased blood perfusion and capillary density.

In conclusion, the present study highlights the dual effects of RWPC on neovascularisation (Fig. 6). RWPC exert their anti-angiogenic activity, at least in part, via an ERα-dependent mechanism. Of particular interest is the demonstration of increased neovascularisation due to a synergistic activation of ERα-dependent VEGF/NO pathway and Sirt-1/AMPKα/PGC-1α/β ERα-independent mechanism. Thus, this study brings a corner stone of a novel pathway for RWPC to correct cardiovascular diseases associated with default of neovascularisation.
